# Pilocarpine as a Purgative for the Horse

**Published:** 1891-12

**Authors:** D. A. Johnson


### PILOCARPINE AS A PURGATIVE FOR THE HORSE.

By Same.

Pilocarpine Hydrochlorate given hypodermically is a good hydragogue purgative for the horse. It is a mild systemic stimulant, excites the activity of the glandular structures, especially the parotid glands and those of the intestinal tract, and stimulates peristalsis, thus causing the discharge of watery fæces.

Thus used it is especially valuable in cases of intestinal impaction, when the alvine excretions are liable to become hard and dry; and in cases where gastric distention has produced intestinal paralysis.

When given hypodermically in doses of one to three grains it should produce a copious flow of saliva in three to five minutes, and an action of the bowels in twenty minutes to one hour, and if it does not the dose should be repeated.

In certain cases it may be well to combine eserine with the pilocarpine, yet much caution should be exercised in the use of this combination, and as eserine is a powerful sedative I have nearly discarded its use.

In comparison the two drugs are as follows:

| PILOCARPINE. | ESERINE. |
|---|---|
| General stimulant. | Powerful sedative. |
| Increases the heart's action. | Decreases the heart's action. |
| Mildly stimulates peristalsis. | Powerful peristaltic stimulant. |
| Lowers temperature. | Increases temperature. |
| Produces watery fæces. | Does not alter the character of the fæces. |

Thus it is seen that eserine is a much more powerful drug than pilocarpine, and will always produce more or less depression; consequently it should never be used when the action of the heart is weak, as is often the case in colic and indigestion.

While in pilocarpine we have a remedy that can be pushed, and in so doing we get the purgative action, and gain two points, *i. e.*, an increased action of the heart, and a lowering of the temperature; and, by its sudorific action it lessens the tendency to congestion of the bowels.

Prof. Niles has demonstrated that eserine, in doses of three grains, will cause the discharge of watery fæces, while smaller doses do not alter the character of the fæces.

